# Healthcare workers' willingness to work during an influenza pandemic: a systematic review and meta-analysis

**DOI:** 10.1111/irv.12310

**Published:** 2015-04-23

**Authors:** Yumiko Aoyagi, Charles R Beck, Robert Dingwall, Jonathan S Nguyen-Van-Tam

**Affiliations:** a Division of Epidemiology & Public Health, University of NottinghamNottingham, UK; b Dingwall Enterprises Ltd, Nottingham Trent UniversityNottingham, UK

**Keywords:** Healthcare worker, pandemic, willingness to work

## Abstract

To estimate the proportion of healthcare workers (HCWs) willing to work during an influenza pandemic and identify associated risk factors, we undertook a systematic review and meta-analysis compliant with PRISMA guidance. Databases and grey literature were searched to April 2013, and records were screened against protocol eligibility criteria. Data extraction and risk of bias assessments were undertaken using a piloted form. Random-effects meta-analyses estimated (i) pooled proportion of HCWs willing to work and (ii) pooled odds ratios of risk factors associated with willingness to work. Heterogeneity was quantified using the *I*^2^ statistic, and publication bias was assessed using funnel plots and Egger's test. Data were synthesized narratively where meta-analyses were not possible. Forty-three studies met our inclusion criteria. Meta-analysis of the proportion of HCWs willing to work was abandoned due to excessive heterogeneity (*I*^2^ = 99·2%). Narrative synthesis showed study estimates ranged from 23·1% to 95·8% willingness to work, depending on context. Meta-analyses of specific factors showed that male HCWs, physicians and nurses, full-time employment, perceived personal safety, awareness of pandemic risk and clinical knowledge of influenza pandemics, role-specific knowledge, pandemic response training, and confidence in personal skills were statistically significantly associated with increased willingness. Childcare obligations were significantly associated with decreased willingness. HCWs' willingness to work during an influenza pandemic was moderately high, albeit highly variable. Numerous risk factors showed a statistically significant association with willingness to work despite significant heterogeneity between studies. None of the included studies were based on appropriate theoretical constructs of population behaviour.

## Introduction

Although variable in severity,^1^,^2^ one consistent feature of pandemic influenza is a surge in demand for health care.^3^,^4^ Hospitalization due to influenza A(H1N1)pdm09 in the USA was estimated at approximately 274 000 cases between April 2009 and April 2010^5^ contrasting with 95 000 annual influenza-associated primary hospitalizations from 1979 to 2001.^6^ In 2009–10, the availability of intensive care unit beds came under pressure in most national health systems.^1^,^7^ Healthcare workers (HCWs) play key roles during an influenza pandemic, but a serious shortage of personnel may occur at peak times or in severe pandemics because of absenteeism due to illness, caring for family members who are ill, or refusal to work.^8^ Effective preparation for the next pandemic requires estimates of HCWs' willingness to work and an understanding of influencing factors.

The available data are highly variable. One Nigerian study found only one quarter of HCWs stating they would be willing to work in a unit treating patients with influenza A(H1N1)pdm09,^9^ whilst an Australian qualitative study of family physicians found 100% of participants willing to work.^10^ Chaffee^11^ first reviewed willingness to work during disasters and reported that the following factors would be influential: type of disaster, concern for close family, friends and pets, responsibility for dependants, the perceived value of one's response, belief in a duty of care, access to personal protective equipment (PPE), provision of basic needs (water, food, rest, shelter and communication tools) and prolonged working hours. Three published reviews reported that similar factors would be associated with willingness to work during an influenza pandemic,^12^–^14^ but the data were not summarized quantitatively.

We addressed this evidence gap by conducting a systematic review and meta-analysis in accordance with the Preferred Reporting Items for Systematic Review and Meta-Analyses (PRISMA) statement. The review questions sought to elucidate the proportion of HCWs willing to work during an influenza pandemic, and to identify risk factors associated with willingness to work. Our findings are interpreted with reference to sociological understandings of population behaviour, which have to date largely been absent from the peer-reviewed literature, but are highly relevant to the development of appropriate interventions to minimize refusal to work.

## Methods

### Search strategy

The study protocol was registered with the National Institute for Health Research international prospective register of scientific reviews (PROSPERO; #CRD42013004865) prior to executing the literature search strategy.^15^ The PRISMA checklist is available as supporting information.

We sought to analyse data collected exclusively from HCWs including doctors, nurses, hospital workers, emergency healthcare service workers, public health workers, medical and nursing students, non-clinical support staff and retirees. The outcome measures of interest were the proportion of HCWs reporting willingness to work during an influenza pandemic, and odds ratios or case counts allowing the derivation of odds ratios pertaining to factors associated with willingness to work. We included study manuscripts written in English reporting original quantitative research derived from a cross-sectional design, studies pertaining to a prior or hypothetical influenza pandemic, and studies reporting data pertaining to the aforementioned outcome measures, with no limitations on the time and place of publication.

The following databases were searched from their inception to April 2013: MEDLINE, EMBASE, Web of Knowledge, SCOPUS, AMED, ASSIA, BioEthicsWeb, CINAHL, Cochrane Library and PsycINFO. Google Scholar and OpenGrey were also searched. Search terms were ‘pandemic + influenza + willingness to work/report to work’ to avoid including studies on willingness to accept vaccination. These terms were used in both keyword and MeSH searches as appropriate for each database as follows: #1. pandemics (MeSH); #02. influenza, human (MeSH); #03. ‘attitude of health personnel’ (MeSH) or willingness (keyword); #04. hospital administration (MeSH) or report to work (keyword); #05. willing* adj5 work (keyword); #06. respon* adj5 work (keyword); #07. would come (keyword); #08. #03 OR #04 OR #05 OR #06 OR #07; #09. #01 AND #02 AND #08 (see also Table S1). Reference lists in eligible articles were also searched.

All identified records were imported to endnote software X4 (Thomson Reuters, Toronto, CA, USA) and duplicate entries removed. The remaining records were screened by a single researcher (YA) against the protocol eligibility criteria following a sequential assessment of the study title, abstract and full-text article. Where this was unclear, agreement on eligibility of each study was achieved through discussion with a second researcher (RD or JSN-V-T). Data extraction was performed by a single researcher (YA) using a piloted form collecting details of study characteristics {title, author, publication year, place, study period, study design, participants, subject [pandemic of avian influenza origin/influenza A(H1N1)pdm09/non-specified, hypothetical influenza pandemic]}; definition of outcome measures; questionnaire type; validation; statistical analysis and any stated limitations; percentage of willingness to work; and risk factors association with willingness. Odds ratios (ORs) of factors both unadjusted and adjusted were extracted to estimate the association with willingness to work. Crude case counts and the percentage of people in each risk factor stratum were extracted where available. Risk of bias was assessed for each study using a Newcastle–Ottawa assessment scale modified for cross-sectional studies by Herzog *et al*.^16^

### Summary measures and analysis

Descriptive statistics were calculated using Microsoft® Office Excel® 2010 (Microsoft Corporation, Richmond, VA, USA). Random-effects meta-analysis estimated the proportion of HCWs (including 95% confidence intervals [CIs]) who reported willingness to work during an influenza pandemic. Random-effect meta-analysis of pooled odds ratios (including 95% CIs) estimated the association of factors with willingness to work.^17^ Heterogeneity between studies was assessed using the *I*^2^ statistic.^18^ We considered it statistically inappropriate to perform meta-analysis where *I*^2^ exceeded 85%.^19^ To explore sources of heterogeneity, we planned to conduct subgroup analyses according to the type of influenza pandemic; geographical region; survey time period; type of questionnaire; type of participants; sex of participants; and Newcastle–Ottawa assessment scale score. We used Galbraith plots to detect those studies that contributed substantial heterogeneity and conducted sensitivity analyses excluding them from our pooled estimates.^20^ For each meta-analysis, publication bias was assessed graphically using a funnel plot of effect size versus standard error and statistically using Egger's regression test.^21^ Meta-analysis of pooled proportions was conducted using statsdirect version 2.7.9 (StatsDirect Ltd., Cheshire, UK), and meta-analysis of pooled odds ratios was conducted using stata® version 11.2 (StataCorp LP, College Station, TX, USA).

## Results

### Study selection

**Figure 1:**
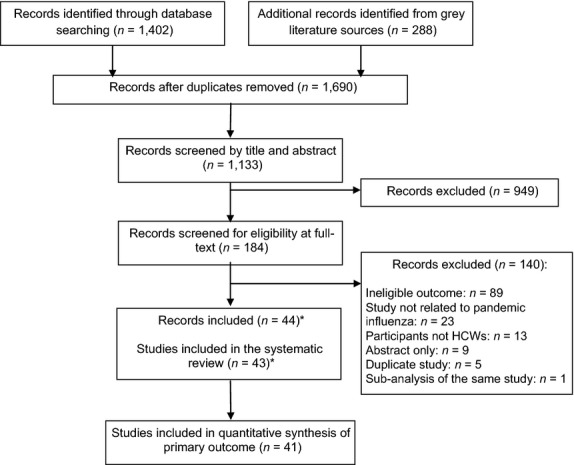
Overview of the selection process. *The findings from one study were reported in two separate papers.^51^,^52^

We identified a total of 1133 unique records of which 43 studies met protocol eligibility criteria (see Figure1). Two studies did not describe the percentage of participants reporting willingness to work; therefore, 41 were included in the meta-analysis of the pooled proportion of HCWs willing to work during an influenza pandemic.

### Study characteristics

The included studies comprised entirely of cross-sectional surveys including two pre-/post-intervention studies and are summarized in Table1. The participant population sizes ranged from 60 to 4306 with a median of 725 (interquartile range [IQR] 308–1711). The earliest publication was in 2006, and the majority of articles were published in 2009 (11; 25·6%) and 2010 (13; 30·2%). 28 of 43 (67·4%) studies used a hypothetical influenza pandemic as the subject, 21 (48·8%) were conducted in the USA, and 21 (48·9%) investigated both clinical and non-clinical staff within hospital settings.

**Table 1:** Characteristics and results of individual included studies

Study	Country	Study period	Participants	Sample size (response rate)	Sampling method	Proportion of HCWs willing to work (95% CI)
Hypothetical scenarios of an influenza pandemic						
Kaiser (2009)^23^	USA	November 2007–March 2008	Medical students	523 (61%)	All medical students in the USA	95·8% (93·7–97·3)
Syrett (2007)^24^	USA	Before 2003	Emergency healthcare department workers	186 (100%)	Convenience (in a medical centre)	77% (70·2–82·7)
Martinese (2009)^25^	Australia	2006 (May–August)	Hospital workers	560 (98%)	Convenience (attendants of meetings)	64·2% (60·2–68·3)
Barnett (2010)^34^	USA	2009 (May–June)	Emergency medical service's workers	586 (49%)	Convenience (responders of other survey)	93·1% (90·8–95·1)
Barnett (2009)^35^	USA	2006–2007	Health department workers	1835 (83%)	Convenience (all employees in four clusters of local health departments)	92% (90·7–93·2)
Errett (2013)^36^	USA	2009 (June–August)	Medical Reserve Corps	3181 (not stated)	Convenience (volunteer group units of which leaders had an interest of the survey)	91·9% (90·9–92·8)
Barnett (2012)^37^	USA	April 2009–June 2010	Local public health department workers	2993 (66%)	Convenience (from 8 local public healthcare departments)	91% (89·9–92·0)
Martin (2011)^38^	USA	2009 (October–December)	Nurses	735 (61%)	Stratified random (from the licence registry list in the region)	90·1% (87·7–92·1)
Stergachis (2011)^39^	USA	2008 (May–November)	Healthcare workers	4306 (50%)	Stratified random (from licence database and hospitals in the region)	89% (88·0–89·9)
Seale (2012)^40^	China	January 2009	Hospital workers	1909 (99%)	Convenience (participants of other RCT in selected wards in 24 hospitals)	86% (84·4–87·5)
Adams (2012)^41^	USA	–*	Healthcare workers	1342 (50%)	Convenience (all staff in selected wards in 6 hospitals)	85·1% (83·1–87·0)
Seale (2009)^42^	Australia	2007 (June–October)	Hospital workers	1079 (90%)	Convenience (all workers in randomly selected wards in two hospitals)	83·3% (81·0–85·5)
Balicer (2010)^43^	USA	2009 (January–March)	Hospital workers	3426 (18%)	Convenience (all employees in a hospital)	82·5% (81·2–83·7)
Barr (2008)^44^	UK	–*	Hospital workers	406 (40%)	Convenience (all doctors, medical students attending a lecture and 500 nurses in a hospital)	79% (74·8–82·9)
Daugherty (2009)^45^	USA	Early 2007	ICU and CCU staff	256 (88%)	Convenience (attendants of meetings in two hospitals)	79% (73·4–83·7)
Cone (2006)^46^	USA	2001–2002	Hospital workers	1711 (85%)	Convenience (from 9 hospitals)	72% (69·8–74·1)
Dickinson (2009)^47^	Canada	2009 (September–November)	Family physicians	192 (22%)	Stratified random (from the list of all family physicians in the region)	71% (63·9–77·2)
Yonge (2010)^48^	Canada	2006 (September)	Nursing students	484 (31%)	Convenience (all nursing students in a university)	67·9% (63·6–72·1)
Stuart (2008)^49^	Australia	2007 (February–April)	Hospital workers	1440 (14%)	Convenience (all staff in a hospital)	67% (64·5–69·4)
Hope (2010)^50^	Australia	2007–2008	Hospital workers	868 (54%)	Convenience (randomly selected from a health service facility)	67% (63·8–70·2)
Gershon (2010)^51^	USA	November 2008–June 2009	Department of health, police, fire, emergency medical services and hospital workers	1103 (42%)	Convenience (six essential organizations including hospital workers and emergency medical service personnel)	66% (63·1–68·8)
Damery (2009)^52^ and Damery (2010)^53^	UK	2008 (July–September)	Hospital workers	1032 (34%)	Convenience (from 3 healthcare trusts)	63% (60·0–65·9)
Gershon (2009)^54^	USA	Not stated	Emergency medical services personnel	129 (not stated)	Convenience (attendants of training programme)	63% at pre-intervention (53·8–71·1%) 66% at post-intervention
Cowden (2010)^55^	USA	2007 (February–June)	Hospital workers	778 (31%)	Convenience (all staff in a hospital)	59·6% (56·1–63·1)
Tippett (2010)^56^	Australia	May 2006	Emergency pre-hospital medical care providers	725 (25%)	Stratified random (from national network of 9 ambulance services)	56·3% (52·6–59·9)
Basta (2009)^57^	USA	2006 (November–December)	Health department workers	2414 (51%)	Stratified random (from 67 county health departments)	56·2% (54·2–58·2)
Balicer (2006)^58^	USA	2005 (March–July)	Health department workers	308 (58%)	Judgement sampling (3 health departments)	53·8% (48·2–59·6)
Hope (2011)^59^	Australia	Late 2008	Senior nurse	60 (93%)	Convenience (attendants of an field exercise from 36 emergency department in the region)	47% at pre-intervention (33·7–60·0%) 82% at post-intervention
Gershon (2010)^60^	USA	2008 (fall)	Home healthcare workers	384 (92%)	Convenience (attendants of training sessions)	43% for current patients (38·0–48·1%) 27% for new patients
Garrett (2009)^61^	USA	2007	Hospital workers	2864 (17%)	Convenience (attendants of focus groups)	Not available; mean willingness score (0–100): 75·6%
Studies of avian influenza						
Butsashvili (2007)^62^	Georgia	During 2003–2007	Hospital workers	288 (not stated)	Convenience (random from selected two hospital)	76% (70·7–80·9)
Bell (2014)^63^	USA	2011 (July–September)	Emergency nurses	332 (46%)	Random (from national database)	84% (79·6–87·8)
Mortelmans (2009)^64^	Belgium	2006 (October–December)	Medical students	243 (30%)	Convenience (all senior medical students in 6 universities)	82·3% (76·9–86·9)
Mitani (2011)^65^	Japan	2008 (September–December)	Hospital workers	1975 (63%)	Convenience (all workers in 6 hospitals)	63·3%; 18·8% unconditionally + 44·5% conditionally (61·1–65·4%)
Tzeng (2006)^66^	Taiwan	December 2005	Nurses	225 (95%)	Convenience (attendants of classes)	56·9% (50·1–63·5)
Irvin (2008)^67^	USA	2006 (July–August)	Hospital workers	169 (90%)	Convenience (attendants of lecture)	50% (42·5–58·1)
Studies of influenza A(H1N1)pdm09						
Wong (2010)^22^	Hong Kong	2009 (June)	Community nurses	401 (67%)	All community nurses in the region	23·1% (19·1–27·6)
Ma (2011)^68^	China	December 2009	ICU staff	695 (95%)	Convenience (21 ICUs)	82·3% (79·3–85·1)
Tebruegge (2010)^69^	Australia	May 2009	Healthcare workers	822 (not stated)	Convenience (selected healthcare interest groups, such as Australian Infection Control Association)	82%; 17·6% unconditionally + 64·4% if treatment or prophylaxis are available (79·2–84·6%)
Kaboli (2010)^70^	Canada	2009 (August–September)	Healthcare workers	4046 (not stated)	All healthcare workers (in all 6 health authorities) in the region	69% (67·6–70·4)
Saleh (2010)^71^	Egypt	After the 2009 pandemic	Nurse and nursing students	256 (not stated)	Convenience (2 hospitals and 2 schools)	58%; 20·6% without any concern and 37·9% with some requests (51·7–63·9%)
Etokidem (2012)^72^	Nigeria	2010	Hospital workers	350 (not stated)	Not stated	25·4% (20·9–30·3)
Imai (2010)^73^	Japan	2009 (June–July)	Hospital workers	1693 (47%)	Convenience (all employees in 3 hospitals)	Not available; 28·4% strong motivation; 14·7% strong hesitation to work

HCW, healthcare worker; ICU, intensive care unit; CCU, critical care unit.

* Study period not specified.

### Assessment of risk of bias

Assessments using the modified Newcastle–Ottawa scale showed that 23 of 43 studies were at moderate risk of bias (2–3 of five stars) for the selection domain, whilst 10 studies were at low risk (4–5 stars) and ten studies were at high risk (0–1 stars); many studies used convenience sampling and few justified the study sample size, appropriately considered non-responders and used a validated measurement tool. For the comparability domain, 24 were at high risk (0 of two stars), eight at moderate risk (one star) and 11 at low risk of bias (two stars). Many studies did not clarify how statistical adjustment for confounding variables was carried out, or reported unadjusted estimates only. For the outcome domain, 39 studies were at moderate risk of bias (two of three stars) and four were at high risk (one star). Willingness to work was self-reported in all 43 studies although the statistical test used was clearly described in only 39 studies (see Figure S1).

### Willingness to work

The percentage of participants who expressed a willingness to work ranged from 23·1% (community nurses during the influenza A(H1N1)pdm09 pandemic in Hong Kong in 2009)^22^ to 95·8% (a study of US medical students targeting a hypothetical influenza pandemic).^23^ We abandoned meta-analysis to estimate a pooled mean proportion of HCWs willing to work due to very high statistical heterogeneity between studies (*I*^2^* *=* *99·2%). Our planned subgroup analyses were unable to adequately explain the sources of heterogeneity between studies as this remained above our threshold of 85% in each analysis. The percentage of willingness to work seemed to depend on the particular context of the study. Studies of hypothetical influenza pandemics, which did not include detailed conditions such as virulence of the strain and availability of protective equipment, tended to show a high level of willingness to work. However, studies of precise scenarios or those which investigated willingness during the relatively mild influenza A(H1N1)pdm09 pandemic tended to present relatively low levels of willingness. This finding may correspond with earlier work by Syrett *et al*.^24^ which showed that willingness to work declined from over 75% to <55% as two simulated mass casualty events progressed and more detailed data became available.

### Factors associated with willingness to work

Data were extracted from 33 studies. Pooled estimates from meta-analyses of individual factors associated with willingness to work are summarized in Table2. Overall, females were one-third less likely to be willing to work compared with males. By occupational group, physicians were most likely to be willing to work, followed by nurses, then other health workers. Urban or metropolitan area workers were less likely to be willing to work than rural area workers. Full-time workers were more likely to be willing to work than part-time employees. Respondents living with children or having childcare obligations were one-third less likely to be willing to work compared with those without these obligations. One study identified that pregnancy in a family member reduced willingness to work.^25^ Marital status (not meta-analysed) did not influence willingness to work.

**Table 2:** Summary of meta-analysis for individual factors associated with willingness to work in included studies

Risk factor	Reference group	Comparator group	Statistical adjustment	Number of studies	Pooled OR (95% CI)	*P* value of OR	*I*^2^ (%)	*P* value of Egger's test
Sex (Female/Male)	3037	8362	Adjusted	8	0·64 (0·50–0·81)	<0·01	63·4	NS
	4440	13 130	Unadjusted	14	0·60 (0·49–0·74)	<0·01	73·1	NS
Doctor/Nurse	134	122	Adjusted	1	–	–	–	–
	5402	2742	Unadjusted	13	1·43 (1·05–1·94)	0·02	78·6	0·04
Nurse/Others	1919	927	Adjusted	2	2·14 (1·43–3·20)	<0·01	20·1	–
	8256	4023	Unadjusted	8	1·56 (1·17–2·08)	<0·01	82·0	NS
Doctor/Others	204	357	Adjusted	2	2·73 (1·37–5·43)	<0·01	29·2	–
	6403	1574	Unadjusted	7	2·43 (1·78–3·31)	<0·01	58·9	NS
Clinical/Non-clinical	964	1622	Adjusted	3	Not valid	–	88·8*	NS*
	2472	4825	Unadjusted	7	Not valid	–	96·1*	NS*
Location (Urban/Rural)	302**	284**	Adjusted	2	0·64 (0·48–0·85)	<0·01	0·0	–
	1078	2776	Unadjusted	2	0·76 (0·61–0·94)	0·01	0·0	–
Employment (Full/Part)	520	4385	Adjusted	3	2·14 (1·58–2·90)	<0·01	6·5	NS
	769	4445	Unadjusted	3	1·76 (1·20–2·57)	<0·01	60·1	NS
Childcare	3650**	2230**	Adjusted	4	0·62 (0·51–0·75)	<0·01	0·0	NS
	7790**	5621**	Unadjusted	11	0·66 (0·56–0·77)	<0·01	43·3	NS
Personal safety	2333**	2855**	Adjusted	5	4·42 (2·89–6·77)	<0·01	68·2	NS
	766	410	Unadjusted	2	3·71 (2·85–4·82)	<0·01	0·0	–
Protective measures	458	410	Adjusted	1	–	–	–	
	458**	410**	Unadjusted	3	Not valid	–	97·8*	NS*
Risk perception	2307**	1987**	Adjusted	6	Not valid	–	88·5*	NS*
	206**	873**	Unadjusted	3	2·27 (1·52–3·41)	<0·01	45·9	NS
Training	1206**	1694**	Adjusted	6	1·38 (1·13–1·68)	<0·01	45·3	0·01
	1966**	1822**	Unadjusted	6	Not valid	–	86·2*	NS*
General Knowledge	2713	4375	Adjusted	5	2·02 (1·31–3·11)	<0·01	83·7	NS
	2801**	2467**	Unadjusted	6	1·78 (1·40–2·26)	<0·01	51·6	NS
Role importance	1750**	1984**	Adjusted	4	4·93 (4·01–6·07)	<0·01	19·0	NS
	737**	439**	Unadjusted	3	Not valid	–	86·2*	NS*
Role knowledge	2498	2517	Adjusted	4	2·66 (1·59–4·45)	<0·01	71·9	NS
	1180**	1277**	Unadjusted	5	2·64 (1·62–4·33)	<0·01	73·5	NS
Confidence in skills	1313**	2699**	Adjusted	4	8·06 (3·35–19·4)	<0·01	74·8	NS
	436**	1018**	Unadjusted	4	4·99 (2·51–9·92)	<0·01	78·9	NS
Pre-experience	284**	117**	Adjusted	2	1·23 (0·93–1·63)	0·14	0·0	–
	603**	493**	Unadjusted	3	1·36 (1·13–1·67)	<0·01	0·0	NS
Confidence in employer	3154**	3721**	Adjusted	8	Not valid	–	86·6*	NS*
	2110**	753**	Unadjusted	5	Not valid	–	85·7*	NS*
Communication skills	2480	2122	Adjusted	3	Not valid	–	96·5*	NS*
	890	286	Unadjusted	2	3·87 (1·26–11·9)	0·02	0·0	NS
Family preparedness	2099	3089	Adjusted	4	Not valid	–	92·6*	NS*
	628	548	Unadjusted	2	Not valid	–	88·3*	–*

OR, odds ratio; CI, confidence interval; NS, not statistically significant.

* Meta-analysis abandoned due to excessive statistical heterogeneity, therefore pooled OR and *P*-value considered invalid, not shown.

** Not all studies provided the numbers of participants in each group.

Perceived personal safety at work and perception of pandemic risk (aware that a pandemic was likely) were both associated with increased willingness to work. Likewise, the provision of protective measures (mainly personal protective equipment) increased willingness to work, although meta-analysis was abandoned due to high heterogeneity (*I*^2^* *=* *97·8%).

Training in pandemic preparedness, general and specific role knowledge, confidence in personal skills, good communication skills and perception of role importance all had positive effects on willingness to work. Confidence in employers as judged by ‘belief that the employer can provide timely information’ also positively influenced willingness to work, although meta-analysis was abandoned due to high heterogeneity.

### Risk of bias across studies

The funnel plot of the percentage of HCWs willing to work did not present a clear funnel shape, appeared to scatter widely without any detectable association with the standard error and overflowed the false 95% CI range. Egger's regression test reached statistical significance and showed that studies reporting a lower percentage were more likely to be published (*P *=* *0·004). Funnel plots and Egger's regressions tests pertaining to meta-analyses of factors associated with willingness to work revealed no evidence of publication bias except for previous training and comparison of physicians and nurses (see Table2), which suggested possible underreporting of studies with an adverse result.

## Discussion

This study advances knowledge from previous reviews on willingness to work during influenza pandemics by adding further new studies and subjecting the findings to statistical evaluation where possible. The search was conducted comprehensively and yielded 43 studies from 11 countries. However, quality of the included studies was not uniformly high and excessive statistical heterogeneity prevented meta-analysis of the primary outcome measure. Whilst it was not possible to identify a single clear source of the heterogeneity encountered, almost certainly the wide variation in settings, scenarios and respondents contributed significantly. Meta-analysis suggested that sex and job category would affect willingness to work although studies varied greatly in the composition of their samples. Hypothetical scenarios varied in virulence, stage and the amount of information provided to respondents. Studies of influenza A(H1N1)pdm09 were conducted at different junctures during the evolution of the 2009–10 pandemic. There was no consistency in terms of how respondents were asked about their willingness to work, and the design of questionnaires used to collect outcome data from respondents varied between studies. Remarkably, despite such high heterogeneity, some factors emerged showing a consistent association with willingness to work. Whilst previous reviews suggested these from a narrative approach, this study has confirmed them statistically.

Being male, a physician or nurse (especially the former), and a full-time worker were all positively associated with willingness to work. These factors are essentially non-modifiable; without access to the raw data, we could not disentangle any potential confounding between being male and the likelihood of being a physician or full-time worker in studies providing only unadjusted ORs. Nevertheless these were consistent findings across most studies and firm knowledge that these are reliable and statistically proven influencers of willingness to work is important information for both policy makers and healthcare service managers, even though they are difficult factors to influence.

Childcare obligation was a consistent barrier to HCWs' willingness to work. The importance of this factor may be an artefact of the high participation of women in the HCW workforce in most countries, combined with traditional cultural expectations that they will take primary responsibility for childcare. It is, nevertheless, an important finding for managers. It is not clear whether this is driven mainly by practicality, that is the need to provide childcare at home, or by concerns about whether the safety of children might be compromised by infection brought in from the parental workplace. Paradoxically, the evidence that HCWs are at increased risk of influenza infection is rather mixed and somewhat inconsistent,^26^ whereas the evidence that children (rather than adults) are usually the introducers of influenza infection into households is firmly established.^27^ This question should be further investigated because it has implications for appropriate organizational responses. If it is simply a practical matter, then managers need to consider what help could be given in emergencies through the expansion of onsite or community childcare provision. If it is a concern about cross-infection, then appropriate education and information programmes may resolve the problem. In either case, it is unlikely that simple disciplinary sanctions will be effective, because of the social force of parental obligations. Indeed, these may well be counterproductive, if other workers perceive them to have been unreasonably applied by managers unsympathetic to real personal dilemmas.

Confidence in safety, risk perception, prior training, general and role knowledge and confidence in skills were statistically proven facilitators for willingness to work. These are all addressable by detailed pandemic preparedness educational activities at healthcare unit level. Importantly, one message arising from assessments of pandemic planning activities prior to the 2009–10 pandemic was that whilst national level pandemic planning was generally successful, the level of planning at local level was insufficient, including training on pandemic influenza for HCWs.^28^ A particular feature of pandemics is the level of anxiety provoked by the disruption of ‘business as usual’ and the destabilization of usually stable organizational environments.^29^ Whilst it is not necessary to retrain HCWs frequently, this is a topic that should be addressed in their basic education and managers should ensure that updating materials are readily available, and regularly revised, so that programmes can rapidly be rolled out when a pandemic is identified. Evidence of organizational preparedness will contribute to the confidence of HCWs that they will not be placed at undue risk by being asked to work in different ways or in different environments from those that they are accustomed to.

A number of limitations with the present study warrant discussion. Our literature search was limited to records published in English. Therefore, we cannot exclude the possibility of having omitted outcome data published in other languages. Many of the included studies were at moderate or high risk of bias. Moreover, only a small number were available for analysis in relation to some risk factors; these results should be interpreted cautiously. The possibility of publication bias might also be a limitation. However, considering that the percentage of willingness was relatively high in most studies, this suggests that unpublished data may not have found statistically significantly higher percentages of willingness to work. Whilst some studies used questionnaires based on recognized psychological theories, these were commonly ‘fear-appeal’ theories. Unfortunately, this may not be appropriate as the preferable behaviour (working during an influenza pandemic) would not result in release from personal fear.^30^ We did not identify any studies that investigated the interaction between individual and organizational responses, which biased the findings towards individual fears rather than the social conditions that might provoke or alleviate these.

As important as our specific results themselves, is the fact that we identified a multiplicity of approaches to studying the issue of HCW willingness to work during a pandemic; mainly small, *ad hoc* enquiries, not based on any consistent scenarios or theoretical approaches. To solve this, a consistent methodological framework is needed before any further studies are undertaken. The outbreaks of Ebola virus disease in West Africa and MERS-CoV in the Middle East offer two very different settings in which to improve study designs and understanding of HCWs' willingness to work where infectious disease creates appreciable personal risk.

In the meantime, policy makers should recognize that HCW willingness to work during an influenza pandemic is likely to be improved by practical measures to support childcare responsibilities and by the timely provision of relevant and high-quality training and information as a pandemic develops. Whilst the above would hold true for influenza, the actual risks and perceptions are not consistent across all novel respiratory viruses. For example, 5% of nurses in Ontario refused to work during the SARS crisis when the risk to HCWs was almost exclusively nosocomial (compared with pandemic influenza where the risk is community-wide).^31^ Similarly, in the ongoing MERS-CoV epidemic, the risk of nosocomial infection is presently greater than in wider community settings.^32^,^33^

## Conclusions

HCWs' willingness to work during an influenza pandemic is moderately high although highly variable, and substantial statistical heterogeneity precluded formal meta-analysis. Numerous risk factors are associated with willingness of HCWs to work during an influenza pandemic, revealing potential points of intervention to increase willingness to work. We identified a wide variety of approaches to the study of willingness to work. For improved future understanding, we advocate a coordinated global approach with standardized protocols and based on appropriate theoretical constructs; and the evaluation of packages of intervention through controlled studies.

## Supporting Information

**Table S1.** Full electronic search strategy (MEDLINE). **Figure S1.** Summary of risk of bias of included studies using the modified Newcastle-Ottawa Scale (*n* = 43).

**Data S1.** PRISMA 2009 checklist.
