# Supplementary material for: Healthcare workers' willingness to work during an influenza pandemic: a systematic review and meta-analysis
Source: Influenza Other Respir Viruses. 2015 Apr 23;9(3):120–30. doi: 10.1111/irv.12310 (PMC4415696; doi:10.1111/irv.12310)

**Healthcare workers’ willingness to work during an influenza pandemic: a systematic review and meta-analysis**

# Supplementary information

# Table S1. Full electronic search strategy (MEDLINE)

| 01. Pandemics (MeSH) |
| --- |
| 02. influenza, Human (MeSH) |
| 03. “Attitude of Health Personnel” (MeSH) or willingness (keyword) |
| 04. Hospital Administration (MeSH) or report to work (keyword) |
| 05. willing* adj5 work (keyword) |
| 06. respon* adj5 work (keyword) |
| 07. would come (keyword) |
| 08. 03 or 04 or 05 or 06 or 07 |
| 09. 01 and 02 and 08 |

## Figure S1. Summary of risk of bias of included studies using the modified Newcastle-Ottawa Scale (n = 43)


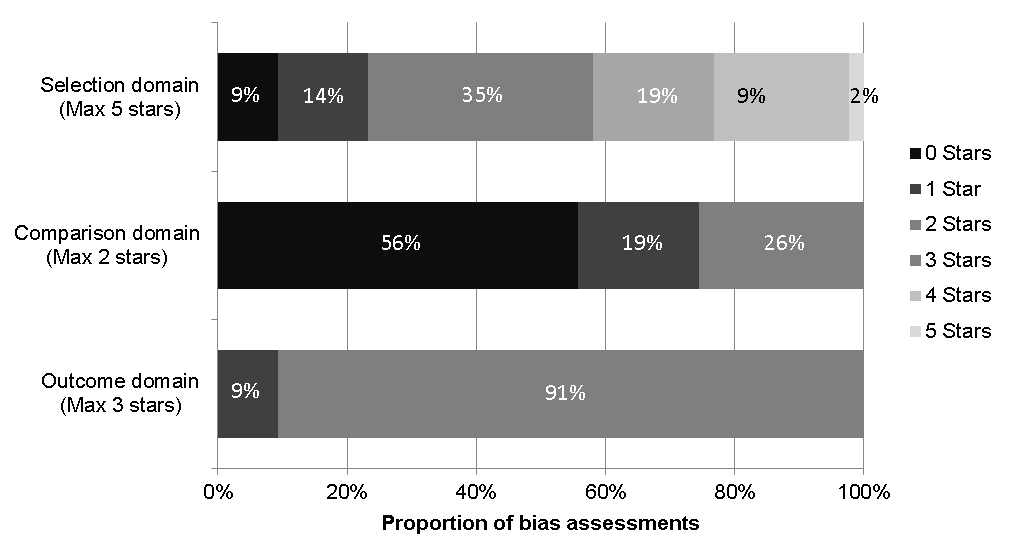

Supplement: Supplementary file 1 [file irv0009-0120-sd1.docx]
